# Interplay between phytohormone signalling pathways in plant defence – other than salicylic acid and jasmonic acid

**DOI:** 10.1042/EBC20210089

**Published:** 2022-09-30

**Authors:** Eleanor Gilroy, Susan Breen

**Affiliations:** 1 Cell and Molecular Science, James Hutton Institute, Invergowrie, Dundee DD2 5DA, U.K.; 2 Division of Plant Sciences, James Hutton Institute, University of Dundee School of Life Sciences, Errol Rd, Invergowrie, Dundee DD2 5DA, U.K.

**Keywords:** crosstalk, immunity, plant hormones, signalling, signalling hubs

## Abstract

Phytohormones are essential for all aspects of plant growth, development, and immunity; however, it is the interplay between phytohormones, as they dynamically change during these processes, that is key to this regulation. Hormones have traditionally been split into two groups: growth-promoting and stress-related. Here, we will discuss and show that all hormones play a role in plant defence, regardless of current designation. We highlight recent advances in our understanding of the complex phytohormone networks with less focus on archetypal immunity-related pathways and discuss protein and transcription factor signalling hubs that mediate hormone interplay.

## Introduction

Plants are sessile organisms, as a result they face varied biotic and abiotic threats. Phytohormones help maintain homeostasis under varied conditions and are essential in fine-tuning growth and immune pathways to best suit the situation. Historically, phytohormones are split into two groups: growth-promoting – gibberellins, cytokinins, auxins, brassinosteroids, and strigolactones; stress/defence-related – ethylene (ET), salicylic acid (SA), jasmonic acid (JA), and abscisic acid (ABA) [1]. However, in recent years, these divisions have become increasingly blurred as more data emerges to suggest that cross-talk between phytohormones is extensive during growth and stress responses. Table 1 overviews phytohormone contributions to abiotic and biotic environmental challenges, but notably these will vary significantly depending on the specific nature of the interaction, e.g. plant/pathogen species, the tissue/organ type, stress intensity and response timeframe examined. This review focuses on recent findings for the roles of hormones in immune signalling and on signalling hubs that act as a site of hormone interplay. In addition, we touch upon how the hormone signalling in immunity and signalling hubs are modified during changing biotic conditions, which will become increasingly important to tackle future climate change. The complexity of SA and JA interplay is covered in another review for this issue. Here, we focus on the more neglected phytohormones on plant pathogen responses and only refer to JA or SA to illustrate their contribution in these interactions. For other recent reviews of hormone signalling and the effects of abiotic stresses, see [2–6].

**Table 1:** How abiotic and biotic environmental stresses influence hormone levels

	Heat	Light	CO_2_	Drought	Flood	Resistance to biotrophs	Resistance to necrotrophs	Resistance to herbivores
**Abscisic acid**	increases [7]	increases [8]	increases [5]	increases [9]	reduces/increases [10]	reduces [11]	increases [11]	reduces/increases [12]
**Auxin**	reduces [7]	reduces [13]	increases [5]	reduces? [6]	reduces [14]	reduces [15]	increases [16]	species/tissue specific [12]
**Brassinosteriods**	increases [17]	reduces [13]	?	reduces [17]	reduces [14]	increases/reduces [18]	increases [18]	reduces [12]
**Cytokinins**	reduces [7]	increases [19]	increases [5]	reduces [19]	reduces [20]	increases/reduces [15]	increases [21,22]	increases [23]
**Ethylene**	increases [24]	reduces [8]	reduces [5]	increases [6]	increases [10]	reduces [25]	increases [26]	reduces/increases [12]
**Gibberellins**	reduces [7]	increases [13]	increases [27]	reduces [6]	reduces [10]	increases [28]	reduces [16]	reduces [12]
**Strigolactones**	increases [29]	increases [8]	?	reduces [6]/increases [29]	?	increases [30]	increases [31]	increases [32]

The table describes how hormone levels can generally be summarized to respond in plants in a range of environmental challenges. However, the responses will vary significantly depending on the specific type of interaction, e.g. plant/pathogen species, the tissue/organ type, stress intensity, and response timeframe examined. The first half of the table summarizes the phytohormone responses during five abiotic stresses. The second half of the table describes how hormones respond in general terms during immune responses induced by biotrophs, necrotrophs, and herbivore pests.

## Molecular interplay of hormones that impacts immunity

Below we have tried to illustrate how the intricate networks of plant responses to phytohormones could impact on immunity through particular examples of relevant molecular interaction. However, most published data are gathered at particular snapshots in time during interactions with each unique trigger and microorganism. Consequently, the quantified signals and responses can vary in timing and intensity. It is also important to emphasize here that phytohormones are often in flux due to a range of external environmental and internal developmental factors and will not act with equal weight, in every tissue type and, at every time point during an infection in regards to their impact on immunity. In addition, there are many mechanisms that can regulate phytohormone activity, e.g. generation or sequestering of bioactives by conjugation and deconjugation all of which are integrally regulated during biotic and abiotic stress.

### Brassinosteroids

**Figure 1:**
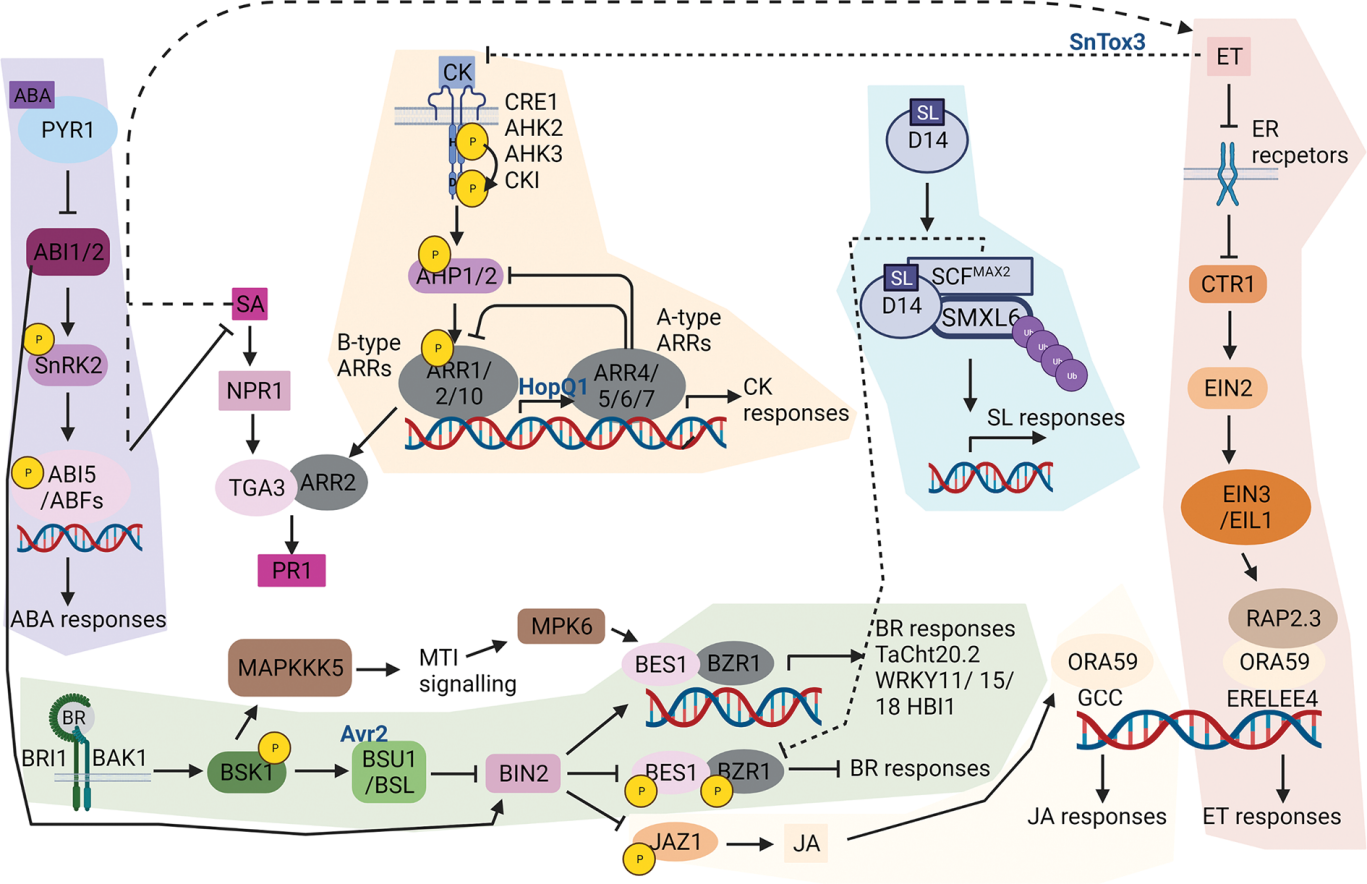
Brassinosteroid, ABA, ethylene, cytokinins, and strigolactones points of cross-talk Brassinosteroid pathway is shaded green. Brassinosteroids (BR) binding to the cell surface receptor BRI1 (BR INSENSITIVE 1) induces association with the co-receptor BAK1 (BRI1-ASSOCIATED KINASE 1). BSK1 (BR SIGNALING KINASE 1) is phosphorylated by BRI1 and interacts with BSU1 (BRI1 SUPPRESSOR 1) family of proteins including BSL1 (BSU1-like 1) [38–41]. BSL1 is a target of the *P. infestans* effector Avr2 [42]. BSU1 dephosphorylates and inhibits BIN2 (BR-INSENSITIVE 2) allowing the transcription factors (TF) BZR1 (BRASSINAZOLE-RESISTANT 1) and BES1 (BRI1-EMS-SUPPRESSOR 1) to induce brassinosteroid response genes and others involved in immunity e.g. Chitinase *TaCht20.2*, and TFs *WRKY11*, *WRKY15*, *WRKY18* and *HBI1* (HOMOLOG OF BEE2 INTERACTING WITH IBH1) [43,44]. In a phosphorylated state, BIN2 phosphorylates BES1 and BZR1 repressing brassinosteroid responses. BIN2 also phosphorylates and destabilizes JAZ1 (JASMONATE ZIM-domain), an inhibitor of JA transcription factors inducing JA responses [37]. BSK1 is also activated by MAMP-triggered immunity, which in turn activates MAPKKK5 (MAPK Kinase Kinase 5) inducing MAMP-triggered immunity signalling [45]. BES1 is a substrate of MPK6 (MAP Kinase 6) induced by MAMP-triggered immunity signalling [46]. ABA pathway is shaded purple. ABA binds to its receptor PYR1 (PYRABACTIN RESISTANCE1), which is up-regulated during biotic stress. This inhibits the activity of ABI1/2 (ABSCISIC ACID INSENSITIVE 1 and 2) thereby activating downstream signalling via phosphorylation of SnRK2 (SNF1-related kinases 2), resulting in phosphorylation of the transcription factorsABI5/ABFs (ABRE-binding factors) inducing ABA responses [11]. Increasing ABA dampens SA signalling and induces ethylene signalling. ABI1/2 de-phosphorylate and inactivate BIN2, activating Brassinosteroid transcriptional responses [34]. Cytokinins pathway is shaded beige. Cytokinins (CK) bind to their receptors; AHK2, AHK3 (HYBRID HISTIDINE PROTEIN KINASE 2, 3), CRE1 (CYTOKININ RESPONSE 1), and CKI1 (CYTOKININ INDEPENDENT 1) that result in phosphorylation of the histidine kinase domain (H) and transphosphorylation of the receiver domain (D). AHPs (histidine phosphotransfer proteins) transmit the signal from the receptor to B-type ARRs (nuclear response regulators). ARR1/2/10 induce the transcription of A-type ARRs (ARR4/5/6/7) to induce cytokinins responses and a negative feedback loop [47]. The effector, HopQ1, from *P. syringae* induces the transcription of A-type ARRs to suppress immunity mediated by FSL2 [48]. Cytokinins and SA act synergistically with ARR2 binding TGA3, to promote the expression of PR-1 [49]. Strigolactones pathway is shaded blue. Strigolactones (SL) binds to DWARF14 (D14) receptor which induces the formation of a protein complex with the negative regulator SMXL6 (SUPPRESSOR OF MORE AXILLARY GROWTH2 1 LIKE) and the F-box protein, SCF^MAX2^ (MORE AXILLARY GROWTH2) resulting in degradation of this complex. This subsequently induces activation of SL responses and transcription of SMXL6, 7, 8 [30,31]. SCF^MAX2^ interacts with BZR1 and BES1 resulting in their degradation [50]. Ethylene pathway is shaded pink. Ethylene receptors are ER localized and in the presence of ethylene (ET) are inhibited, reducing the activity of a Raf-like serine/threonine (Ser/Thr) kinase, CTR1 which in turn activates the Nramp metal ion transporter family protein EIN2 (ETHYLENE-INSENSITIVE 2), allowing transcription factors; EIN3, EIL (EIN3-like), and ERFs (ETHYLENE RESPONSE FACTORS) to induce transcription [51]. The ERF Family Transcription Factors, OCTADECANOID-RESPONSIVE ARABIDOPSIS 59 (ORA59) and RAP2.3 interact to mediate ethylene signalling [52]. During ethylene signalling ORA59 binds the ERELEE4 promoter element however when induced by JA it binds a GCC box promoter element to induce JA transcriptional activation [53]. The authors acknowledge that all known and emerging interaction events could not be included in this figure created with BioRender.com.

Brassinosteroids are classically thought of as growth-promoting. However, they have varying outcomes on immunity during diverse plant/pathogen interactions and at different developmental stages, though generally, biotrophic pathogens show reduced disease severity upon BR treatment [18,33]. The BR pathway has many links to microbe-associated molecular pattern (MAMP)-triggered immunity and many other hormone pathways, depicted in Figure 1. Further downstream, BR-INSENSITIVE 2 (BIN2) a GLYCOGEN SYNTHASE KINASE 3 (GSK3)/shaggy-like kinase family member acts as a negative regulator of BR signalling and a regulation hub for modifying growth and defence responses. BIN2 is regulated by many proteins. The negative regulators of ABA, ABA INSENSITIVE 1 and 2 (ABI1 and ABI2) are protein phosphatase 2Cs that dephosphorylate and inactivate BIN2, resulting in accumulation of dephosphorylated BRI1-EMS-SUPPRESSOR 1 (BES1), a transcriptional activator of brassinosteroid transcriptional responses [34]. Intriguingly, ABI1 inhibition of BIN2 is repressed during drought stress, allowing BIN2 to phosphorylate and stabilize the NAC transcription factor, RESPONSIVE TO DESICCATION 26 (RD26) to promote drought stress tolerance [35]. ABI genes are up-regulated by effector secretion in the *Arabidopsis* *thaliana*-*Pseudomonas syringae* pathosystem [36], suggesting an additional function for ABA-brassinosteroid-mediated BIN2 regulation during abiotic stress. BIN2 is also a negative regulator of resistance against the fungal pathogen, *Verticillium dahlia*, as loss of BIN2 increased resistance. BIN2 also phosphorylates and destabilizes JASMONATE ZIM DOMAIN 1 (JAZ1), an inhibitor of JA responses [37], highlighting how brassinosteroids can influence JA-dependent immune responses.

The brassinosteroid response transcription factors, BES1 and BRASSINAZOLE-RESISTANT 1 (BZR1), represent another point of immune regulation. BES1 is a substrate of Arabidopsis MAP kinase 6 (MPK6) linking brassinosteroids with-MAMP-triggered Immunity signalling, increasing resistance to hemibiotrophic bacterial pathogens but conversely increasing susceptibility to necrotrophic pathogens [54,46]. BZR1 induces the expression of WRKYs (WRKY11, WRKY15, and WRKY18) and the bZIP HOMOLOG OF BEE2 INTERACTING WITH IBH 1 (HBI1) transcription factor, which negatively modify immunity [44,55]. Moreover, wheat BZR2 is upregulated in seedlings during treatment with two defence elicitors, flg22 and Pst322 [43]. Consequently, wheat overexpressing *TaBZR2* demonstrated increased resistance to *Puccinia striiformis* f. sp. *tritici* and an increase in chitin-binding and chitinase activity [43]. Furthermore, TaBZR2 functions in drought tolerance [56] enhancing the evidence that biotic and abiotic regulation is tightly linked.

### Abscisic acid

Abscisic acid is a sesquiterpene found in plants, fungi and bacteria [57–59]. In plants, ABA primarily modifies responses to abiotic stress like drought and salinity; however, it’s induction of stomatal closure and increasing callose deposition are important against pathogen invasion [60,61]. ABA has an antagonistic relationship with SA, thus increasing susceptibility to biotrophic pathogens (infect living host tissue). ABA has a synergistic relationship with ethylene (Figure 1), resulting in increased resistance to necrotrophic pathogens (actively kill host tissue), via receptor PYRABACTIN RESISTANCE 1 (PYR1) [11]. Consequently, ethylene accumulation may not be a direct result of ABA acting on the ethylene pathway but indirectly by SA repression facilitating ethylene signalling. ABA/PYR1-induced activation of SnRK2 dampens SA-mediated resistance, confirming previously published data that *P. syringae* modulates host ABA biosynthesis, to down-regulate SA biosynthesis and SA-mediated defences to aid disease progression [11,36,62]. Furthermore, increased ABA concentrations increased susceptibility of barley to *Magnaporthe oryzae* and wheat to *Fusarium graminearum*, both hemibiotrophic fungi [63,64].

Exogenous ABA application induces expression of three protein phosphatases 2C (PP2Cs) genes, known as HIGHLY ABA-INDUCED PP2C GENEs (*HAI1, HAI2*, and *HAI3)*. All interact and inactivate the pathogen/elicitor-activated MAP kinases, MPK3 and MPK6, resulting in ABA-mediated immune suppression [65]. However, mutants lacking these PP2Cs have increased ABA levels, and increased susceptibility to *P. syringae*, while an ABA biosynthetic mutant, with reduced ABA levels, shows enhanced disease resistance [36,62]. This was further supported by hormone profiling of resistant and susceptible kiwifruit during challenge with *P. syringae*. ABA concentration was significantly reduced in resistant kiwifruit due to down-regulation of biosynthesis genes and up-regulation of ABA catabolism genes [66]. Concomitantly, JA and SA levels remained unchanged in resistant kiwifruit but were significantly increased in susceptible plants where ABA remained stable [66]. The ability of bacterial and fungal pathogens to generate ABA, which can negatively affect plant defences, indicates that pathogens can utilize ABA as an effector to modulate immunity. Moreover, ABA is crucial to managing trade-offs between biotic and abiotic stress responses by integrating conflicting signals differently in young and old leaves; SA mediated defences are up-regulated in young leaves while ABA-mediate immune suppression occurs in old leaves [67].

### Ethylene

Ethylene is a gaseous hormone, detected by multiple types of ethylene receptors on the endoplasmic reticulum. In the presence of ethylene, these receptors are inhibited, reducing the activity of the protein kinase, CONSTITUTIVE TRIPLE RESPONSE 1 (CTR1), allowing de-repression of ETHYLENE INSENSITIVE 2 (EIN2) and multiple transcription factors like EIN3, ETHYLENE-INSENSITIVE3-LIKE 1 (EIL), and ETHYLENE RESPONSE FACTORS (ERFs) to induce ethylene responses [51]. Plants overexpressing ERF-transcription factor OCTADECANOID-RESPONSIVE ARABIDOPSIS AP2/ERF 59 (ORA59) showed an increase in ethylene sensitivity and resistance to *Pectobacterium carotovorum*, a necrotrophic bacterium [52]. Interestingly, ORA59 is also involved in JA transcriptional regulation by binding a GCC box promoter element when induced by JA and to the ERELEE4 promoter element when induced by ethylene, thus mediating hormone signalling from two hormones to induce defence signals [53] (Figure 1).

Ethylene is involved in all levels of immune signalling from colonization, MAMP-triggered immunity and effector triggered immunity (ETI). EIN2 mediates defence signalling in response to *Phytophthora infestans* reducing pathogen penetration thus, ethylene is required for pre-invasion defence [68]. Flg22 treatment of leaves induces the accumulation of ethylene, JA, and SA. However, *A. thaliana* plants insensitive to ethylene cannot produce reactive oxygen species (ROS) and nitric oxide (NO) after flg22 treatment [69]. In rice, the resistance protein Pik-H4, interacts with the homeodomain type transcription factor BTH-induced homeodomain protein 1 (OsBIHD1) to modify the ethylene and brassinosteroid pathways during *rice blast* disease [70]. *OsBIHD1* knockout plants had reduced resistance to *M. oryzae* whereas over-expression lines were dwarfed, insensitive to brassinosteroids, showed up-regulated ethylene synthetic gene expression and more resistant to *M. oryzae* [70]. Another resistance protein, RPW8.1 (RESISTANCE TO POWDERY MILDEW 8.1) binds ACO4 (1‐AMINOCYCLOPROPANE‐1‐CARBOXYLATE OXIDASE 4) which increases ethylene signalling, however ACO4 and other proteins, ETHYLENE RESPONSIVE ELEMENT BINDING FACTOR 6 (ERF6) and ORA59, feedback to negatively regulate RPW8.1 resulting in reduced expression and reduced resistance [71].

The ethylene pathway is targeted by many effector proteins from various pathogens to modify immune signalling. Soybean, GmACS (1-AMINOCYCLOPROPANE-1-CARBOXYLATE SYNTHASE) is targeted by PsAvh238, from *Phytophthora sojae*, disrupting ethylene biosynthesis and ethylene -induced resistance [72]. ACO1 from *Pinus thunbergia* is targeted by BxSCD1, an effector from the pine wood nematode, *Bursaphelenchus xylophilus* that suppresses cell death induced by a *B. xylophilus* MAMP [73]. During *B. xylophilus* infection, PtACO1 is up-regulated. When the BxSCD1 effector is silenced, PtACO1 expression is reduced, indicating that BxSCD1–PtACO1 interaction modifies ethylene biosynthesis and plant defence [73]. The EAR-motif (ethylene-responsive element binding factor-associated amphiphilic repression motif) containing effector, Jsi1 (JASMONATE/ETHYLENE SIGNALLING INDUCER 1), from *Ustilago maydis* interacts with members of the TPL/TPR (TOPLESS/TOPLESS RELATED) family, most likely activating the ERF branch of JA and ethylene signalling to increase biotrophic susceptibility [74]. Effectors from other fungi, *M. oryzae, Sporisorium scitamineum*, and *S. reilianum*, also contain EAR-motifs and interact with TPL/TPR proteins [74].

### Gibberellins

**Figure 2:**
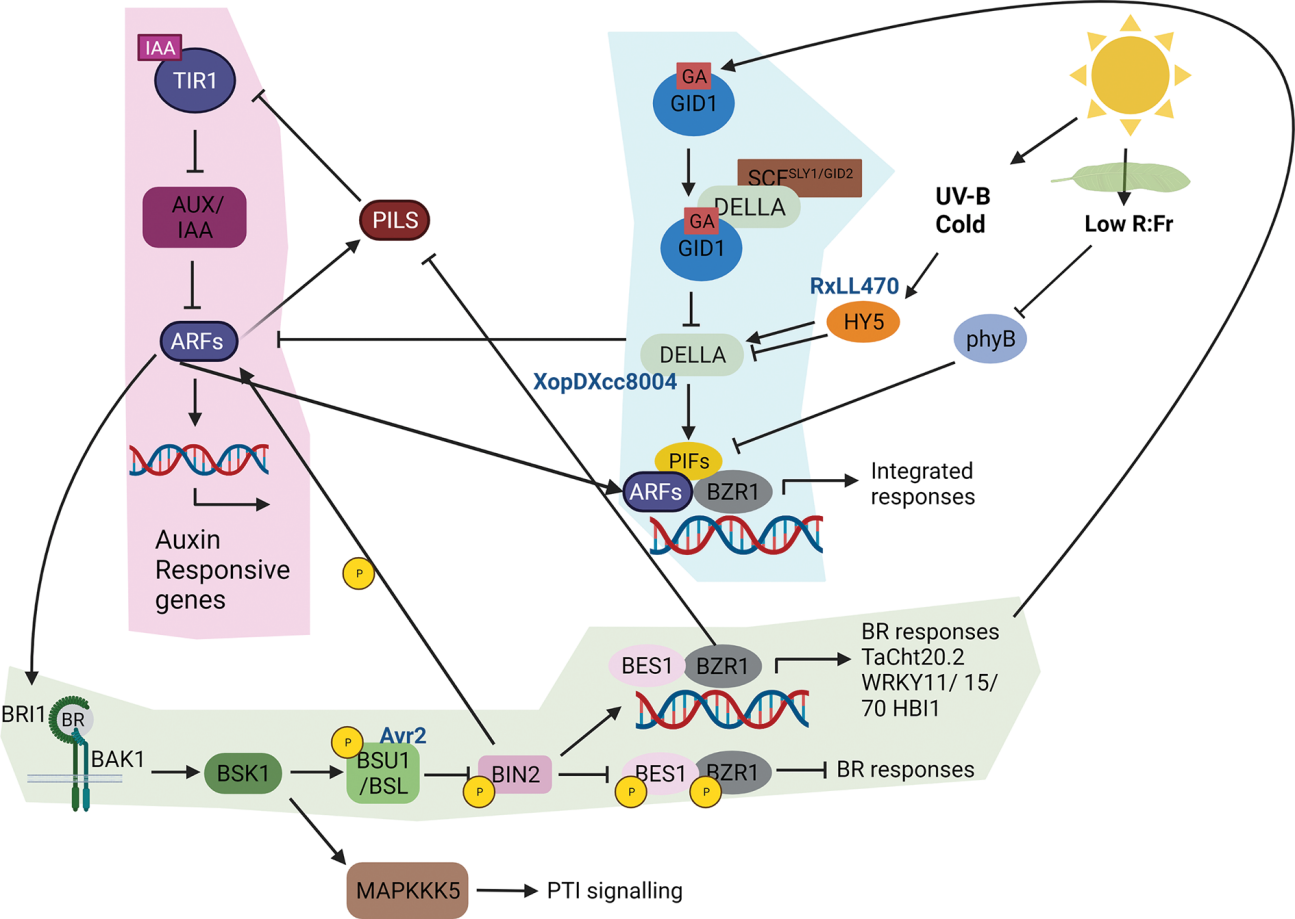
Brassinosteroid, IAA, giberellins, and light points of cross-talk The SCF^TIR1/AFB^-dependant auxin response pathway is shaded in pink. The TIR1/AFB auxin co-receptors belong to a family of F-box proteins and mediate responses after perceiving auxin. Binding IAA triggers recruitment of the SCF^TIR1^ E3 ubiquitin protein ligase complex to the Auxin/Indole-3-Acetic acid (Aux/IAA) repressor proteins causing their degradation. This allows Auxin response factor (ARF) transcription factors to dimerize at the promoters of auxin-inducible genes thus activating gene expression [78]. ARFs can interact with transcription factors from other pathways such as PIF4 (PHYTOCHROME INTERACTING FACTOR 4) and BZR1. Auxin modulates the brassinosteroid pathway via BRI1 which has an auxin-response element targeted by ARFs [79]. Auxins increase expression of brassinosteroid receptor, BRI1, and brassinosteroid -responsive genes due to the presence of auxin-response elements targeted by ARFs in their promoters [79]. The RXLR effector PSE1from *Phytophthora parasitica* reduces auxin accumulation by altering distribution of the PIN4 and PIN7 auxin efflux carriers making the plant more susceptible [80]. The Brassinosteroid pathway is shaded green and the described brassinosteroid pathway components are same as in Figure 1. Brassinosteroid responses can promote gibberellin biosynthesis to increase DELLA degradation [81]. PIN-LIKES (PILS) protein family of auxin transport facilitators can control the intracellular auxin accumulation at the ER regulating auxin homeostasis [79]. Brassinosteroid signalling restricts PILS transcription and protein levels and, thereby, increases nuclear abundance and signalling of auxin [79]. BIN2 is crucial to potentiate auxin signalling by phosphorylating ARF7 and ARF19 [82]. However, de-phosphorylation of BIN2 during immune responses reduces auxins signalling. The Gibberellin pathway is shaded in blue. Gibberellins (GA) activates receptor GID1 (GIBBERELLIN INSENSITIVE DWARF1) which induces interaction with DELLA proteins [76]. The GA-GID1-DELLA complex interacts with SCF^SLY1/GID2^, a E3 ubiquitin ligase, and is targeted for proteasomal degradation [77,83] resulting in a lower abundance of DELLAs. DELLAs are negative regulators of many transcription factors that can heterodimerise to induce integrated responses e.g. from light, brassinosteroid and auxin response pathways [84,85]. The *Xanthomonas campestris* effector protein, XopD_Xcc8004_, targets and delays the degradation of a GA repressor DELLA protein to trigger plant disease [86]. When leaves are shaded from light, then a low Red:FarRed causes accumulation of the inactive form of the red light receptor Phytochrome B (PhyB) which suppresses the activity of PIFs [78]. UV-B and Cold stress activate the positive bZIP transcriptional regulator, HY5 (ELONGATED HYPOCOTYL 5). HY5 promotes the accumulation of DELLA proteins under UV-B radiation [87] while cold stress, can activate or suppress DELLAs, which control a multitude of other responses relevant in plant immunity [88] as described in Figure 3. The Arabidopsis downy mildew effector HaRxLL470 suppresses plant immunity by decreasing the DNA‐binding activity of HY5 [89]. The HOMOLOG OF BEE2 INTERACTING WITH IBH1 (HBI1), functions with other transcription factorsto mediate positive and negative transcriptional changes of many growth and defence responses [55,90]. The authors acknowledge that they couldn’t include all known and emerging interaction events in this figure. Created with BioRender.com.

**Figure 3:**
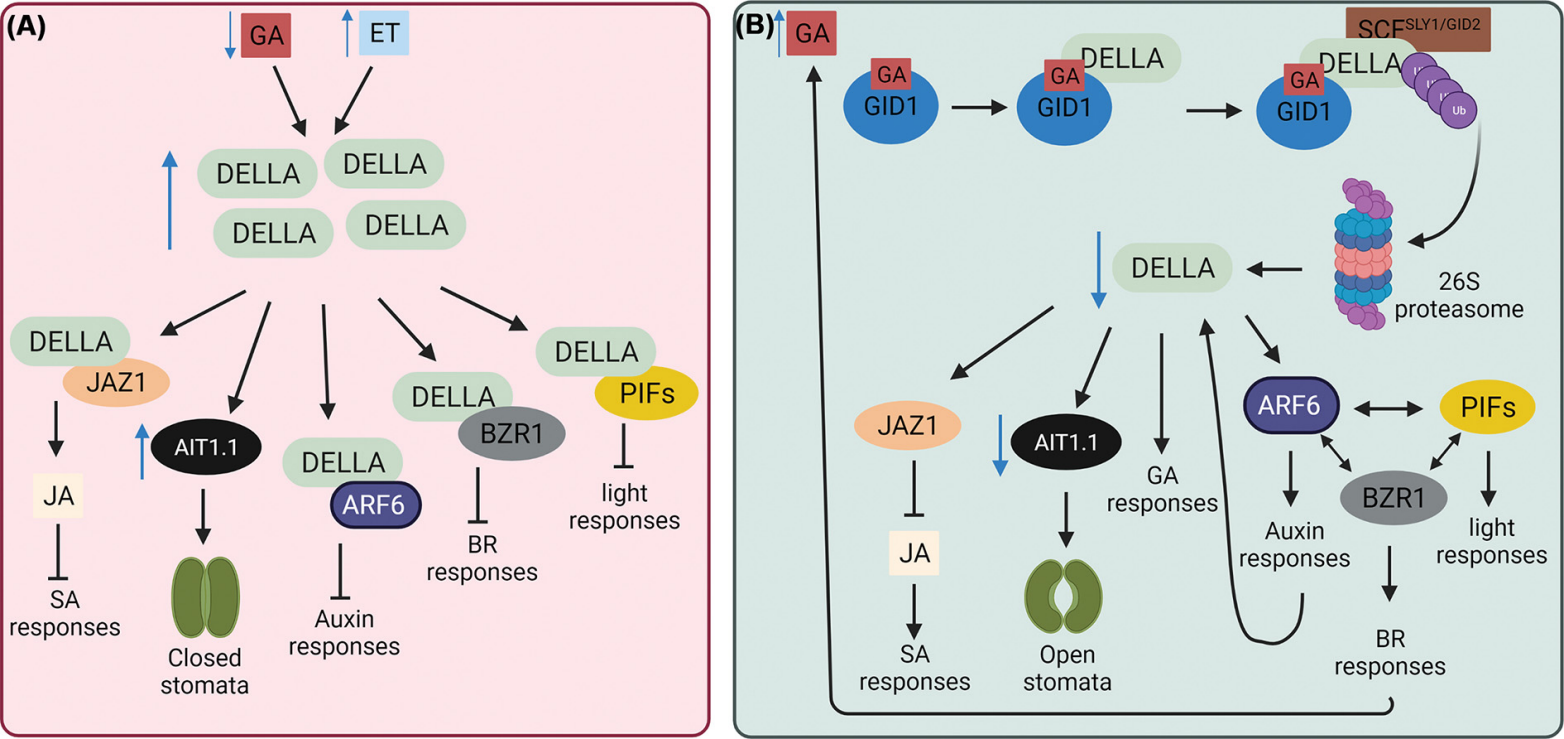
DELLA signalling hub (**A**) Low gibberellins (GA) hormone levels and high ethylene (ET) levels result in a high abundance of DELLA proteins allowing them to bind transcription factorsfrom many pathways. The abundance of DELLAs can result in inhibition or induction of other hormone signalling depending on the function of the targets. The DELLA–JAZ1 interaction results in increased JA signalling and decreased SA [134]. The tomato DELLA, PROCERA, up-regulates the ABA transporter AIT1.1, promoting ABA-induced stomatal closure [135]. DELLA–ARF6 interaction results in inhibition of Auxin signalling [84]. DELLA–BZR1 interaction results in inhibition of brassinosteroid signalling while the DELLA–PIF interaction results in inhibition of light responses [85]. (**B**) An increase in gibberellins (GA) hormone levels results in gibberellins binding its receptor GID1 that induces interaction with DELLA proteins [76]. The GA–GID1–DELLA complex interacts with SCF^SLY1/GID2^, a E3 ubiquitin ligase, and is targeted for proteasomal degradation [77,83] resulting in a lower abundance of DELLAs. This reduction in DELLAs initiates transcription of gibberellin response genes [85] while also releasing JAZ1, a repressor of JA signalling which results in an increase of SA signalling [129]. It also down-regulates AIT1.1 increasing stomatal opening [130] and releases the TFs BZR1, PIFs and ARF6 allowing mediation of brassinosteroid and Auxin signalling along with light responses [125]. Brassinosteroidscan also promote gibberellins biosynthesis to increase DELLA degradation [81] while auxin biosynthesis and transport controls DELLA protein abundance [84]. The authors acknowledge that all known and emerging interaction events could not be included in this figure. Created with BioRender.com.

Gibberellins were first identified as a growth regulators in the 1980s [75]. GIBBERELLIN INSENSITIVE DWARF1 (GID1) is the nuclear localized gibberellin receptor [76] which in the presence of gibberellins, binds DELLA proteins, negative regulators of gibberellin signalling, forming a GA–GID1–DELLA complex that is degraded resulting in gibberellin mediated signalling (Figures 2 and 3) [77]. This induces large scale changes in other hormone signalling pathways and so the role of DELLAs as a signalling hub will be discussed later.

Although, gibberellins are primarily growth hormones, during *Pestalotiopsis versicolor* infection (Twig blight pathogen) of bayberry there is significant differentiation of gibberellin-responsive genes between resistant and susceptible cultivars [91] indicating that the regulation of gibberellins is important for resistance. Plants infected with the phloem colonising bacteria *Candidatus Liberibacter asiaticus* (CLas) show ROS production, including H_2_O_2_, and ROS-producing NADPH oxidases [92]. ROS levels during infection can be reduced by addition of NADPH oxidase inhibitor, diphenyleneiodonium (DPI), indicating the importance of NADPH oxidases during infection [92]. Exogenously applied gibberellic acid induces the gene expression of H_2_O_2_-scavenging enzymes and reduces NADPH oxidases as well as increasing immunity to CLas [92]. Exogenous gibberellin, GA3 also enhanced resistance of rice to plant hopper, *Nilaparvata lugens* (Stål) [93]. This increased resistance was associated with the decrease in OsMPK6 and OsWRKY13, OsWRKY30, and OsWRKY33, leading to reduced JA levels but increased ethylene levels [93].

ELONGATED HYPOCOTYL 5 (HY5) is a bZIP transcription factor acting downstream of multiple photoreceptors and cold responses, reducing growth by reducing GA3 levels while also regulating strigolactones (see section below) [88, 94, 95]. HY5 influences the abundance of gibberellins by regulating the accumulation of DELLA proteins under low intensity and long wavelength UV-B conditions (Figure 2) [87]. Interestingly, an effector (RxLL470) from *Hyaloperonospora arabidopsidis* perturbs the DNA-binding activity of HY5, resulting in suppressed immunity [89]. Rice infected with the necrotrophic fungi *Fusarium fujikuroi*, show abnormal growth phenotypes, due to the secretion of a gibberellin mimic from the fungi, which aids in disease development [96]. These data suggest pathogens use gibberellin to manipulate both growth and defence regulation.

### Strigolactones

Strigolactones are carotenoid-derived plant hormones that are biosynthesised in plastids [97]. Strigolactones modulate growth and development during a range of environmental stresses e.g. regulating stomatal aperture in response to drought, darkness and high CO_2_ [98] which also protects against air pollutants and pathogen invasion [99]. Strigolactones and ABA have similar chemical structures and are both derived from the carotenoid pathway, so their biosynthesis is often coregulated [100]. Interestingly, the effect of Strigolactones metabolism on ABA homeostasis are opposite in roots and shoots under stress [30].

Strigolactones regulate immune and growth responses and have an indirect influence on the activities of other hormones and key components of signalling cascades of other growth regulators [30,31]. Cytokinins and strigolactones act as negative and positive regulators, respectively, in many plant drought responses. Strigolactones play an important role in defence as a counter measure to bacterial cytokinins produced by the leaf gall forming bacteria, *Rhodicoccus fascians* [99]. The exogenous application of strigolactones induce several auxin responsive genes and the ROS- and SA-inducible glutaredoxin [98]. Strigolactones also play a positive role in tomato immunity to *Meloidogyne incognita*, a root-knot nematode, as loss of strigolactones biosynthetic genes reduces immunity while exogenous application of strigolactones enhanced immunity [101]. During strigolactone-activated defence there is cross-talk between JA and ABA pathways potentially mediated by the MYC-RELATED TRANSCRIPTIONAL ACTIVATOR (MYC2), a crucial regulator of JA signalling during development and stress responses. MYC2 was up-regulated in a strigolactones biosynthetic gene mutant and had a negative effect on defence [101]. The E3 ubiquitin ligase subunit, MORE AXILLARY BRANCHES 2 (MAX2), initially characterised during plant development, plays an essential role in strigolactones perception and acts as a key hub to mediate immunity, e.g. *max2* plants show increased susceptible to *P. syringae* [98,102]. In addition, the F-box domain of MAX2 targets the chaperonin-like SMAX1-LIKE proteins (SMXLs) for degradation [103]. SMXLs are up-regulated by strigolactones and may function like the repressor DELLA proteins, as a loss-of-function mutant result in a constitutive strigolactone response. Evidence suggests that MAX2 can directly interact with and cause the degradation of BZR1 and BES1 from the BR pathway (Figure 1) and acts in a parallel signalling pathway to the guard cell ABA signalling pathway [98,50].

### Auxins

Auxins are well-known regulators of growth and development and an area of keen interest due to there multifunctionality in regulating trade-offs with defence suppression. The major bioactive form is indole-3-acetic acid (IAA), and its biosynthesis, concentration and distribution are tightly regulated [104,105]. Signalling is fine-tuned in various tissues through auxin response transcription factors (ARFs). ARFs also act as central hubs of co-expression, as most Aux/IAA protein and class A ARFs can interact with each other and with multiple transcription factors from other pathways (Figure 2) [106]. DELLA proteins interact with and target ARFs for degradation, while auxin biosynthesis and transport controls DELLA protein abundance (Figure 3) [84,107] suggesting a complex interaction between auxins and gibberellins. Auxins cross-talk with other growth-promoting hormones, although less is known for interactions with strigolactones and cytokinins during immunity.

Auxins are known to increase expression of the brassinosteroid receptor, BRI1, and brassinosteroid-responsive genes through ARF-binding auxin-response elements in their promoters (Figure 2) [79]. BIN2 is also crucial to potentiate auxin signalling by phosphorylating the AUXIN RESPONSE FACTORs, ARF7 and ARF19 (Figure 2) [82]. De-phosphorylation of BIN2 during immune responses reduces auxin signalling. Auxin signalling and plant growth are inhibited by MAMP-triggered immunity [108] and exogenous SA treatment triggers the stabilization of Aux-IAA protein that negatively regulate auxin signalling, thus attenuating auxin responses. Meanwhile, activation of auxin signalling can suppress SA biosynthesis and SA signalling. In addition, Arabidopsis plants defective in auxin signalling or treated with auxin transport inhibitor TIBA (2,3,5-triiodobenzoic acid) have been shown to be more susceptible to the necrotrophic fungus *Plectosphaerella cucumerina* [109].

Changes in auxin signalling can increase susceptibility to infection explaining why several pathogens synthesise auxins, trigger or manipulate auxin signalling in the host as a means of increasing virulence. Bacterial and fungal pathogens can produce auxins to promote infection including *P. syringae* and *Leptosphaeria maculans* [110–112]. Mutation of the bacterial auxin biosynthetic pathway may affect pathogen growth while exogenous application of indole-3-acetic acid induces expression of virulence factors, such as type III secretion system effectors and enzymes involved in cell wall degradation [110,113]. Pathogens also target the auxin pathway using effectors e.g. PENETRATION SPECIFIC EFFECTOR 1 (PSE1) from *Phytophthora parasitica* reduces auxin accumulation at the root apex. This alters distribution of the PIN-FORMED auxin efflux carriers, PIN4 and PIN7, making the plant more susceptible to disease and suppressing immune responses [80]. In addition, several different rice RNA viruses manipulate auxin levels by targeting components of the auxin signalling pathway facilitating infection and resulting in dwarf phenotypes [114–116].

### Cytokinins

Cytokinins play important roles in mediating plant growth, promoting cell division, preventing senescence and regulating biotic and abiotic stress tolerance [117,47]. In addition, cytokinins positively promote plant immunity against infection by pathogens of various lifestyles [23,118–120]. Cytokinins mediate resistance against *P. syringae* in tobacco through increased antimicrobial phytoalexin synthesis, independent of SA signalling [121]. Cytokinins also act synergistically with SA signalling to promote resistance by activating many ARABIDOPSIS RESPONSE REGULATORS (ARR). Indeed, ARR2 binds SA activated transcription factor, TGA1A-RELATED GENE 3, (TGA3), to promote the expression of the SA-responsive gene PATHOGENESIS-RELATED 1 (PR-1) (Figure 1) [49]. Cytokinins’ interaction with auxin is implicated in immune responses, early signalling, and deciding the fate of plant–microbe interactions. Contrasting responses induced by auxins and cytokinins modulate plant–pathogen interactions during infection of Arabidopsis by *P. syringae* [122]. In fact, *P. syringae* enhances auxin concentrations but downregulates cytokinin biosynthesis genes to decrease cytokinin levels [123]. Many microbial plant pathogens, secrete cytokinins analogues or activate plant cytokinins production to divert nutrients from the host. Therefore, pathogen-derived cytokinins have been shown to mediate host susceptibility possibly via exploiting cytokinins control over senescence. One example is the production of three cytokinins by the bacterial phytopathogen *Rhodococcus fascians* to maintain tissue proliferation in infected areas [78]. Furthermore, many biotrophic and hemibiotrophic fungi produce cytokinins to promote localised host tissue viability resulting in green island disease symptoms [78]. In addition, the *P. syringae* effector HopQ1 activates the type A ARRs, increasing cytokinins signalling to suppress immunity mediated by FLAGELLIN-SENSITIVE 2 (FLS2) [15,48]. The effector SnTox3 from the necrotrophic pathogen *Stagonospora nodorum* reduces accumulation of cytokinins by glycosylation, oxidative degradation and inhibition of biosynthesis in an ethylene dependant manner (Figure 1) [22]. Cytokinins can also induce systemic immunity in tomato by regulating the trafficking of the tomato PRR ETHYLENE INDUCING XYLANASE 2 (LeEIX2) that recognizes the Xyn11 family of xylanases [21]. This recognition induces resistance against *Botrytis cinerea* and *Oidium neolycopersici* in an SA- and ethylene-dependent manner [21].

## Hubs of interplay that impact on immunity

### Transcription factors

Transcription factors play a crucial role in mediating hormone cross-talk between growth, development, and defence responses. In this section, we will provide examples that demonstrate this best. The brassinosteroid pathway transcription factor, BZR1 forms a regulatory BAP module (BZR1–ARF6–PIF4 complex) with multiple transcription factors from other pathways for example, auxin responsive ARF6 and the negative regulator of phytochrome signalling pathway, PHYTOCHROME-INTERACTING FACTOR 4 (PIF4), which regulates multiple growth signals to control gene expression under varying environmental stresses [13,124,125]. These individual transcription factors share significant overlap in the genes they regulate, e.g. ARF6 modifies expression of almost half the genes targeted by BZR1 and PIF4 [125]. Interestingly, under low gibberellin conditions these transcription factors are inhibited by DELLAs, resulting in JA signalling [17].

PIFs are a cross-talk point between light, JA and ethylene signalling in immunity. PIFs mainly function as negative regulators of photomorphogenesis in presence of red light but also negatively regulate defences e.g. against the necrotrophic pathogen *Botrytis cinerea* in a CORONATINE INSENSITIVE 1 (COI1)- and EIN2-dependent manner [126]. Expression of defence response genes, ERF1, ORA59 and PLANT DEFENSIN 1.2 (PDF1.2), was induced in *pif1/3/4/5* mutants and lower in *PIF-*overexpressing plants [126]. While PIF4/5 bind the promoter of ERF1, overexpression of ERF1 rescued the increased susceptibility observed in *PIF-*overexpressing plants [126]. This shows the interplay in TF control between biotic and abiotic stress.

MYC2 is a well-known, JA transcription factor that mediates signalling cross-talk between most phytohormones. MYC2 is up-regulated by both JA and ABA and induces signalling from both pathways, whereas it suppresses ethylene responses by downregulation of the transcription factor ORA59 [127–129]. MYC2 is mediated by DELLAs, so only fully active when JA and gibberellins are present [130]. MYC2 also directly binds the promoters of some auxin-responsive transcription factors to repress them, thus affecting the expression of several PIN auxin-efflux carriers and influences auxin-induced susceptibility [131,132]. Furthermore, ARF2, the pleiotropic regulator of auxins and negative regulator of ABA, up-regulates PLETHORA1 (PLT1) which is a integrase-type DNA-binding superfamily protein, thus uncovering a complex transcriptional regulatory architecture surrounding the JA, ABA and auxin response pathways that impact on immunity [133].

### DELLAs

DELLA proteins are nuclear transcriptional regulators, also known as growth repressors, that mediate hormone cross-talk with gibberellins, brassinosteroids, ABA, ethylene, and JA (Figure 3). Given the importance of the DELLA proteins, their transcription is tightly regulated by transcription factors from many hormone signalling pathways including EIN3, CBF1 and JIN1/MYC2, ethylene and JA signalling, respectively [85]. DELLAs are also regulated by a light associated transcription factor, PIF3-like 5 (PIL5), highlighting the complexity of mediating growth, biotic, and abiotic signals [85].

DELLA proteins were initially identified as negative regulators of gibberellin signalling [136] and contain a GA3 perception region, that facilitates binding to GID1 [76]. The GA–GID1–DELLA complex interacts with a ubiquitin E3 ligase complex, SCF^SLY1/GID2^ and is targeted for proteasomal degradation [77,83]. It is the abundance or degradation of DELLAs that swings the complex hormone balance within a plant to facilitate growth- or defence-related signal transduction. The gibberellin dependant degradation of DELLAs induce gibberellin response genes while DELLA accumulation, without gibberellins, inhibits gibberellin responses. Interestingly, gibberellin signalling and biosynthetic mutants, with increased DELLA abundance, show more susceptibility to hemibiotrophic bacteria and increased resistance to necrotrophic fungi [28]. Therefore, the function of GA-DELLAs is important to mediate defence signals. DELLAs are positive regulators of JA as DELLAs bind JAZ1, a negative regulator of JA, therefore gibberellins and JA, via DELLAs, are antagonistic [134]. Increased gibberellins, decrease DELLA abundance, resulting in stronger repression of JA response, resulting in increased SA responses. ABA signalling is also mediated by DELLAs. The tomato DELLA, PROCERA, upregulates the ABA transporter ABA-IMPORTING TRANSPORTER 1.1 (AIT1.1), promoting ABA-induced stomatal closure which restricts pathogen entry. Gibberellins signalling is also affected by ethylene which increases DELLA abundance [85,135]. In plant immunity, a bacterial effector, XopD_Xcc8004_ from *Xanthomonas campestris* blocks GA-DELLA degradation allowing increased pathogenicity [86].

DELLAs contain other conserved domains, including LHR1 (leucine heptad repeats) which is essential for interaction with transcription factors e.g. PIFs and BZR1 [85]. Interaction of DELLAs with BZR1 block DNA-binding thus inhibiting expression of brassinosteroid target genes. Increasing gibberellins, reduces the DELLA-BZR1 interaction, increasing brassinosteroid target genes expression that can attenuate MAMP-triggered immunity [85,90]. However, brassinosteroidscan also promote biosynthesis of gibberellins to increase DELLA degradation, increasing BZR1 activity [81]. Furthermore, the NAC transcription factor JUNGBRUNNEN1 (JUB1) represses *GA3ox1* and *DWF4*, gibberellin and brassinosteroid biosynthetic genes respectively, causing accumulation of DELLAs and increased susceptibility to *P. syringae* [137].

Interestingly, DELLAs can be targeted for degradation, in a gibberrelin-independent manner, by the E3 ubiquitin ligase, CONSTITUTIVE PHOTOMORPHOGENIC1 (COP1) [138]. COP1 destabilization of DELLAs is in response to increased temperature and shade [138], thus providing evidence that the DELLAs are an important signalling hub to regulate biotic and abiotic signals.

## Concluding remarks

Fundamental research is continuing to unravel the complex interplay between plant hormones that regulated growth, development, and immunity during biotic and abiotic stress conditions; however, a clear ‘map’ of all interactions is still a work in progress. However, with the improvement of growth facilities and imaging technologies, these will help dissect more fine detail of the interplay between pathways. In addition, the cross-talk of hormones during biotic and abiotic stress will be a key area in future research with increasing changes to global climates which impact not only plant growth but also their exposure to new and varied pathogens. As an example, ABA, known for its abiotic stress tolerance will become increasingly important for agriculture in a warming world; however, ABA’s ability to suppress immunity may have significant impact on the prevalence of crop disease. Therefore, research into varying environmental conditions combined with pathogen infection will be crucial for the future of the growth, resilience and resistance in our favourite crop varieties. What is clear from the work detailed above is that all hormones affect both growth and immunity.

## Summary

- Most phytohormone signalling pathways contribute, directly or indirectly, to the outcome of plant–microbe interactions.
- Manipulating any one of these hormone pathways for crop improvement for a changing climate could have far reaching consequences on the traits controlled by multiple pathways.
- Fundamental science continues to play an essential role in dissecting the functions of many of the key components and identifying cross-talk hubs of phytohormone pathways.
- Applying this fundamental knowledge in crop plants will allow us to best manipulate pathways and key components for plant immunity in a changing climate.
- There is still some way to go to possess a clear ‘map’ of the phytohormone pathways and what we could achieve through gene editing for improved growth, resilience and resistance in our favourite crop varieties in the field.

## Open Access

Open access for this article was enabled by the participation of University of Dundee in an all-inclusive *Read & Publish* agreement with Portland Press and the Biochemical Society under a transformative agreement with JISC.

## Abbreviations

ABA: abscisic acid
ARF: auxin response transcription factor
ET: ethylene
GA: gibberellins
IAA: indole-3-acetic acid 2
JA: jasmonic acid
PP2C: protein phosphatase 2C
SA: salicylic acid
TIBA: 2,3,5-triiodobenzoic acid
